# Sensitivities of conventional and new electrophysiological techniques in carpal tunnel syndrome and their relationship to body mass index

**DOI:** 10.1186/1749-7221-4-12

**Published:** 2009-07-31

**Authors:** Recep Aygül, Hızır Ulvi, Dilcan Kotan, Mutlu Kuyucu, Recep Demir

**Affiliations:** 1 Department of Neurology, Atatürk University Faculty of Medicine, 25240 Erzurum, Turkey

## Abstract

The purpose of this study is to evaluate prospectively the sensitivities of conventional and new electrophysiological techniques and to investigate their relationship with the body mass index (BMI) in a population of patients suspected of having carpal tunnel syndrome (CTS).

In this study, 165 hands of 92 consecutive patients (81 female, 11 male) with clinical diagnosis of CTS were compared to reference population of 60 hands of 30 healthy subjects (26 female and 4 male). Extensive sensory and motor nerve conduction studies (NCSs) were performed in the diagnosis of subtle CTS patients. Also, the patients were divided into subgroups and sensitivities were determined according to BMI.

The mean BMI was found to be significantly higher in the CTS than in the control group (*p *< 0.001). The sensitivity of the median sensory nerve latency (mSDL) and median motor distal latency (mMDL) were 75.8% and 68.5%, respectively. The most sensitive parameters of sensory and motor NCSs were the difference between median and ulnar sensory distal latencies to the fourth digit [(D4M-D4U), (77%)] and the median motor terminal latency index [(mTLI), (70.3%)], while the median-to-ulnar sensory action potential amplitude ratio (27%) and the median-thenar to ulnar-hypothenar motor action potential amplitude ratio (15%) were least sensitive tests. Sensory tests were more sensitive than motor NCSs. Combining mSDL with D4M-D4U, and mMDL with mTLI allowed for the detection of abnormalities in 150 (91%) and 132 (80%) hands, respectively. Measurements of all NCSs parameters were abnormal in obese than in non-obese patients when compared to the BMI.

The newer nerve conduction techniques and combining different NCSs tests are more sensitive than single conventional NCS test for the diagnosis of suspected CTS. Meanwhile, CTS is associated with increasing BMI.

## Introduction

The carpal tunnel syndrome (CTS), caused by compression of the median nerve at the wrist, is considered to be the most common entrapment neuropathy in adults, with a 10% lifetime risk in the general population [1]. Conventional electrophysiological studies have been useful in the diagnosis of this condition [2-5]. Electrophysiological testing remains an essential technique for quantifying median nerve function in CTS due to its inherent reliability, reproducibility, and objectivity. Nerve conduction studies (NCSs) measure peripheral nerve function directly without subjective bias and without contamination by central nervous system pathways [6]. Median sensory and motor NCSs confirm a clinical diagnosis of CTS in patients with a high degree of sensitivity and specificity [4]. Many authors have attempted to determine the sensitivity of the various tests for early diagnosis of CTS, but it is unclear which is the best.

The median sensory nerve latency (mSDL) in wrist-digit segment was being widely accepted as a more sensitive parameter than median motor studies in detecting early CTS, because CTS usually presents with sensory symptoms and sensory fibers are often affected first [2,4]. However, comparison of the sensitivities of the sensory NCSs techniques had demonstrated that the mSDL is less sensitive than techniques which evaluate median-ulnar sensory latency differences in the same hand [7,8]. On the other hand, median motor distal latency (mMDL) has been reported to be abnormal in 20–81% of patients, depending upon the severity of CTS in the population investigated [3,4].

Comparative tests of sensory nerve latency between the median nerve and the ulnar nerve are well documented in the literature [8,9]. Also, comparative studies between median and ulnar motor latencies with thenar and hypothenar recording have been described, but have not been widely adopted during clinical testing for CTS due to a low diagnostic sensitivity [10,11]. However, the median-thenar to ulnar-hypothenar motor latency differences (M-U LD) may be advantageous when a concomitant polyneuropathy is present, and they may also help to avoid technical pitfalls. Even if the median sensory action potential amplitude (mSNAPa) could not be evoked, median compound muscle action potential amplitude (mCMAPa) might be recordable in numerous patients with severe CTS.

Basing a diagnosis of CTS on a single test comparison, particularly a diagnosis for which surgical intervention may be considered, is potentially problematic, especially when abnormalities are not marked (9). Combining sensory and motor NCSs may allow for the detection of abnormalities in CTS, and may yield a markedly improved diagnostic rate compared with mSDL or mMDL alone.

On the other hand, obese populations are especially susceptible, given that CTS is associated with an increased body mass index (BMI) [12]. The relationship between BMI and CTS could be explained either due to increased fat deposition in the carpal canal or higher hydrostatic pressure in the carpal tunnel in obese subjects [13].

The purpose of this study is, firstly, to evaluate prospectively the sensitivities of conventional and new electrophysiological techniques in a population of patients suspected of having CTS, and secondly to compare the sensitivities of single nerve conduction tests with combining multiple tests. In addition, we investigate their relationship to the BMI. Thus, extensive sensory and motor NCSs were performed in the diagnosis of subtle CTS patients.

## Methods

### Patients and Controls

Ninety-two consecutive patients, ranging in age from 18 to 72 years (mean ± SD: 45.7 ± 10.4 years; 81 female, 11 male), with the clinical diagnosis of CTS, were prospectively evaluated. In the analysis all affected hands were included. Also, thirty healthy subjects were assessed with bilateral NCSs. The aim and methods of the study were explained to all patients and controls before their informed consent was obtained. The medical history and symptoms of CTS such as tingling, numbness, paraesthesia and/or pain for at least 3 months in all or part of the hand territory innervated by the median nerve, mainly at night or on waking and/or triggered by certain postures or repetitive forced movements of the fingers or wrist were included [14]. Meanwhile, the patients were divided into subgroups and sensitivities were determined according to BMI. They were classified as non-obese (BMI < 30) or obese (BMI ≥ 30). BMI was calculated as weight/height^2 ^(kg/m^2^).

Inclusion criteria were as follows: (a) The symptoms occurring longer than 3 months before the study; (b) No corticosteroid injection or carpal tunnel release for CTS before the study; (c) No clinical or electrophysiological evidence of accompanying conditions that could mimic CTS or interfere with its evaluation such as cervical radiculopathy, or significant polyneuropathy.

### Electrophysiological study

Electrophysiological studies were performed according to the American Association of Electrodiagnostic Medicine guidelines [2-5] with a Medelec Teca Premerie Plus vE05 electromyograph (Surrey, UK) in all subjects by the same person. All tests were done in similar temperature conditions, and when the hands were cold they were warmed-up. Skin temperature on the hand was measured and maintained at or above 32°C.

Recorded parameters included:

• Motor NCSs: mMDL, median motor nerve conduction velocity (mMNCV), mCMAPa, median motor terminal latency index (mTLI), M-U LD, and the median-thenar to ulnar-hypothenar motor CMAPa ratio (M/U CMAPa ratio);

• Sensory NCSs: mSDL, median sensory nerve conduction velocity (mSNCV), mSNAPa, the difference between median and ulnar sensory distal latencies to the fourth digit (D4M-D4U), the difference between sensory median distal latencies to second digit and ulnar distal latencies to fifth digit (D2M-D5U), and the median-to-ulnar SNAPa ratio (D2M/D5U SNAPa ratio).

NCSs were done using standard techniques of supramaximal percutaneous stimulation and surface electrode recording. The electroneurographic recordings of motor conduction velocity were made with the filter bandpass at 2 Hz to 3 kHz, a sweep speed of 2 ms/division, and the amplifier gain at 2–4 mV/division. For measurement of sensory action potential amplitude (SNAPa), the instrument settings were: filters, 20 Hz to 3 kHz; sweep, 2 ms/cm; gain, 10–20 μV/division.

Compound muscle action potentials amplitude (CMAPa) and latency were recorded from the abductor pollicis brevis for the median nerve and the adductor digiti minimi for the ulnar nerve. The mMDL was measured from the onset of the stimulus artifact to the onset of the CMAPa. The mTLI was calculated as follows: terminal distance ÷ (mMNCV × mMDL) (4). The distal distance (approximately 7 cm) for the CMAPa was measured in a straight line between the distal stimulation site at the proximal wrist crease and the center of the recording disc electrode.

SNAPa studies were performed to the second, fourth and fifth digits. For both the median and ulnar nerves the wrist stimulation points were 14 cm proximal to the recording electrode. SNAPa latency and amplitude were obtained antidromically and recorded by ring electrodes placed at the proximal and distal interphalangeal joints of the second digit for the median nerve and the fifth digit for the ulnar nerve. Distal sensory latencies were measured from the onset of the stimulus artifact to the peak of the SNAP.

Electromyographic examination was performed on the abductor pollicis brevis muscle using a monopolar needle electrode. Special attention was given to the presence of spontaneous activity at rest.

We considered the mSDL (normal value ≤ 3.5 ms) and the mSNCV (normal value ≥ 40 m/s) or the mMDL (normal value ≤ 3.77 ms) as the main tests for CTS diagnosis. A secondary criterion was the comparison of the D4M-D4U. In our study a D4M-D4U above to 0.45 ms was considered abnormal. The abnormal cutoff values for these parameters were calculated as *plus or minu*s 2 standard deviations from the mean values of control group. A comparison with the ulnar nerve in the same hand was done in each case.

### Statistical Analysis

For statistical analysis, we used the SPSS package 10.0.7 for Windows XP. The mean, standard deviation (SD) and range were calculated in control and CTS groups for each parameter. In the analysis all affected hands were included. mSNAPa or mCMAPa responses which were absent were not included in the mean and standard deviation calculations, but were included in the sensitivity calculations. Student's t test was used to compare the differences among the values between groups. Diagnostic sensitivity of each parameter was determined from CTS group. The sensitivity of each subtest for CTS was calculated as: (number of hands with positive test and CTS ÷ number of hands with clinical CTS) × 100. Meanwhile, sensitivities were determined and compared according to the BMI with the Chi-square tests. Correlations between the electrophysiological parameters with symptom duration and BMI were analyzed using Pearson's correlation coefficient. A probability (*p*) value of less than 0.05 was considered significant.

## Results

### Controls

Thirty healthy subjects (60 hands; 26 female and 4 male) ranged in age from 18 to 62 years with a mean age of 44.3 ± 8 were evaluated with bilateral NCSs, including all electrophysiological parameters. The mean (range) of the D4M-D4U, mSDL, mMDL and mTLI were 0.16 ± 0.14 (0.0–0.45), 3.06 ± 0.22 (2.7–3.5), 3.07 ± 0.35 (2.3–4) and 0.41 ± 0.038 (0.33–0.56) in the control group, respectively. Comparing the median sensory latency to digit IV with the ulnar sensory latency to the same digit, the difference (D4M-D4U) was 0.30 msec or less in 91.7% of the hands. Distribution of all NCSs parameters were normal range. The detailed demographic, clinical characteristics and NCSs parameters are compared in Table 1, 2 and 3.

**Table 1:** Demographic characteristic of patient and control groups

	Controls	CTS	p
**Sex**			
**Female, n (%)**	26 (86.7)	81 (88.1)	NS
**Male, n (%)**	4 (13.3)	11 (11.9)	
**Age (years), mean **± **SD**	44.3 ± 8	45.7 ± 10.4	NS
**BMI, mean **± **SD**	26.5 ± 3.6	29.1 ± 4.8	0.000
**Symptom duration (months), mean **± **SD**	-	35.9 ± 38.2	-

*NS*: non-significant, *SD*: standard deviation

**Table 2:** Shows the mean values, standard deviations and range of the conduction values in controls and CTS groups.

	Controls (n = 60)		CTS (n = 165)		Mean ± 2 SDs of Controls	p*
	Mean ± SD	Range	Mean ± SD	Range		
**D4M-D4U (ms)**	0.16 ± 0.14	0.0–0.45	1.39 ± 1.12	0.31–5.7	0.45	0.000
**D2M-D5U (ms)**	0.18 ± 0.13	0.02–0.45	1.06 ± 0.83	0.24–4.1	0.45	0.000
**D2M/D5U SNAPa ratio (μv)**	1.21 ± 0.36	0.61–2.3	0.80 ± 0.41	0.14–2.2	0.50	0.000
**mSDL (ms)**	3.06 ± 0.22	2.7–3.5	4.04 ± 0.88	3.5–7.2	3.50	0.000
**mSNCV (m/s)**	46.5 ± 2.91	40–52	36.1 ± 7.2	19.4–39.3	40.6	0.000
**mSNAPa (μv)**	26 ± 9.0	10.8–44	16.3 ± 9.4	2.7–36	8	0.000
**mMDL (ms)**	3.07 ± 0.35	2.3–4	4.6 ± 1.6	2.5–11.2	3.77	0.000
**mMNCV (m/s)**	58.5 ± 3.1	52.2–67	56.4 ± 5.1	32.7–68	52.2	0.002
**mCMAPa (mv)**	11.4 ± 3.2	5.2–20	8.7 ± 4.0	0.2–19	5	0.006
**mTLI**	0.41 ± 0.038	0.33–0.56	0.29 ± 0.08	0.12–0.50	0.33	0.000
**M-U LD (ms)**	0.35 ± 0.32	-0.4–1.0	1.67 ± 1.58	-0.1–8.23	0.99	0.000
**M/U CMAPa ratio (mv)**	0.85 ± 0.25	0.33–1.78	0.72 ± 0.36	0.01–1.99	0.35	0.021

*n*: number of hands; *SD*: standard deviation; *CTS group value vs. controls

**Table 3:** Comparison of subtest sensitivity for diagnosis of CTS

	Criteria for abnormality	% Sensitivity of abnormal value	No. of abnormal hands
**D4M-D4U (ms)**	> 0.45	77	127
**D2M-D5U (ms)**	> 0.45	73.3	121
**D2M/D5U SNAPa ratio (μv)**	< 0.50	27	45
**mSDL (ms)**	> 3.5	75.8	125
**mSNCV (m/s)**	< 40	75.8	125
**mSNAPa (μv)**	< 8	38.2	63
**mMDL (ms)**	≥ 3.8	68.5	113
**mMNCV (m/s)**	≤ 52	20.6	34
**mCMAPa (mv)**	< 5	21.6	35
**mTLI**	< 0.33	70.3	116
**M-U LD (ms)**	> 1.0	66	109
**M/U CMAPa ratio (mv)**	< 0.35	15	25

### Patients

Ninety two patients (165 hands) with a clinical diagnosis of CTS were compared to a reference population of 60 hands from 30 controls. The detailed demographic and clinical characteristics are compared in Table 1. Although, the age and sex of the subjects were not significantly different between the CTS and the control groups (p > 0.05), the mean BMI (26.5 ± 3.6 vs 29.1 ± 4.8; t = 3.5; *p *< 0.001) was found to be significantly higher in the CTS than in the control group. The duration of symptoms was between 4 and 240 months (mean ± SD: 35.9 ± 38.2). CTS was severe in 35 (21%) hands of patients. There were 21 (60%) hands with severe CTS cases, in which a median mSNAPa could not be evoked. NCSs showed 2 persons with absent motor responses in patients with severe CTS. Table 2 shows the mean and standard deviation of the NCSs values in two groups. Overall, there were significant differences in all nerve conduction parameters among the two groups when data were corrected for age (*p *< 0.005).

The sensitivity for the diagnosis of CTS for each subtest is shown in Table 3. The most sensitive parameters of sensory and motor NCSs were the D4M-D4U (77%) and mTLI (70.3%), while the D2M/D5U SNAPa ratio in sensory and M/U CMAPa ratio in motor NCSs was least sensitive tests. However, sensory parameters were more sensitive than motor NCSs. Every hand with an abnormal mSDL had an abnormal sensory D4M-D4U. In approximately 20.6% of patients, mMNCV is slowed in the forearm, usually in association with prolongation of the mMDL. The sensitivity of an abnormality of the mCMAPa was 16.5%. The results revealed that measurement of mTLI and mMDL as well as comparison of mSNCV in the carpal tunnel with that in the forearm had approximately similar diagnostic sensitivity while D4M-D4U demonstrated higher abnormality. Combining mSDL and D4M-D4U, D4M-D4U and D2M-D5U or mMDL with mTLI and mMDL with M-U LD allowed for the detection of abnormalities in 150 (91%), 149 (90%) or 132 (80%) and 131 (79.4%) hands, respectively.

Measurements of all NCSs parameters were abnormal in obese than in non-obese patients when compared to BMI. The sensitivity of the D4M-D4U, mSDL, mMDL and mTLI were 81.8%, 80%, 70.2% and 72.6% in obese patients, and 73%, 71%, 65.4% and 67.9% in non-obese patients, respectively. Of the other parameters, the D2M-D5U had the highest sensitivity in obese and non-obese patients. However, statistically significant differences were not found in sensitivities of NCSs parameters between two groups when compared to BMI.

After correlation analysis, none of these parameters were associated to the duration of disease of the CTS subjects. A significant negative correlation was found between the BMI with mSCNV (*r *= -0.21, *p *= 0.012), mSNAPa (*r *= -0.22, *p *= 0.009) and mMNCV (*r *= -0.24, *p *= 0.002). But, there was a positive correlation between the BMI and mMDL (*r *= 0.20, *p *= 0.011). There was no correlation between the BMI with other NCSs parameters. A strong positive correlation was found between mSDL with mSNAPa (*r *= -0.61, *p *= 0.000), and between mMDL with mCMAPa (*r *= -0.39, *p *= 0.000). There was no correlation between forearm mMNCV and mTLI (*r *= 0.013; *p *= 0.87), although mTLI was strongly correlated with mMDL (*r *= -0.86, *p *< 0.000), indicating a disproportionate conduction across the carpal tunnel (data not shown).

## Discussion

NCSs are commonly used in the assessment of patients with numbness, tingling and pain in the hands. CTS is one of the most common disorders for which NCSs are performed. A variety of sensitive NCSs are available for the evaluation of a patient with suspected CTS [1-4]. Unfortunately, no consensus exists regarding the type and number of nerve conduction tests needed to establish the neurophysiological diagnosis in CTS [15]. The AAEM practice parameters for electrodiagnostic studies in CTS reported the sensitivities of the conventional tests to be 56% to 85%, with specificities of 94% or greater [3,4].

In this study, the sensitivity of the mSDL and mMDL were 75.8% and 68.5%, respectively. The most sensitive parameters of sensory and motor NCSs were the D4M-D4U (77%) and mTLI (70.3%), while the D2M/D5U SNAPa ratio (27%) in sensory and M/U CMAP ratio (15%) in motor NCSs were least sensitive tests. However, sensory parameters were more sensitive than motor NCSs. Usually; isolated abnormalities of median motor nerve conduction with normal median sensory NCSs are not due to CTS, extra care is required to exclude other causes, such as radiculopathy [2]. Median sensory and motor NCSs are valid and reproducible laboratory studies that confirm the clinical diagnoses of CTS with a high degree of sensitivity and specificity. Previous publications involving the electrodiagnosis of CTS have reported a wide range of results for the sensitivity of mMDL (20% to 81%) [16,17], wrist-digit sensory latency (40% to 100%) [18,19], and of median-ulnar sensory latency difference (56% to 100%) [3,4,8,20]. Presumably, the wide variation in the number of positive studies is the result of selection factors.

In this study, the results showed that measurement of mSDL as well as comparison of mSCNV in the CTS had similar diagnostic power while D4M-D4U demonstrated higher accuracy. Because fibers from the fourth digit may be more susceptible to compression due to the position of ring finger fibers in the outer margin of the median nerve just beneath the transverse carpal ligament [2]. Every hand with an abnormal mSDL had an abnormal sensory D4M-D4U. In addition to mMDL and mSDL measurements, several new tests have been successively introduced to improve the sensitivity of NCSs (2). Measurement of median-ulnar comparison has been considered superior to mMDL and mSDL measurements, particularly in detecting patients with mild CTS [7]. Subtle abnormalities in CTS may be demonstrated by comparison of the findings in the median nerve with a normal nerve.

Our study demonstrated that the mSNAPa was lower sensitivity than the mSDL (38.2% versus 75.8%). Kuntzer [21] confirmed that the measurements of median sensory conduction from digit to wrist are more often abnormal than the measurement of mSNAPa, 49% versus 30%. Measurement of mMDL was more abnormal than the measurement of mCMAPa (68.5% versus 21.6%), but not the mTLI (70.3%) in this study. Another study showed 15% of mCMAPa were abnormal, similar to our result [21], because the amplitude of the median-thenar response was abnormal only in cases of severe CTS. In 20.6% of patients, mMNCV was slightly slowed in the forearm, usually in association with prolongation of the mMDL. The cause of the slowing of median motor conduction in the forearm of CTS patients is not clear. Wilson [22] provided evidence that the measured slowing is due to the block of conduction of the faster conducting fibers at the wrist. However, Chang et al [23] denied the role of selective conduction block of the large fibers and suggested that the slowing is due to retrograde axonal atrophy of motor fibers in the forearm segment of the median nerve.

When compared to single nerve conduction tests, combining multiple test results has been shown to be superior for diagnosing CTS in other published studies [9-11,24]. In this study, combining mSDL and D4M-D4U, D4M-D4U and D2M-D5U or mMDL with mTLI and mMDL with M-U LD allowed for the detection of abnormalities in 150 (91%), 149 (90%) or 132 (80%) and 131 (79.4%) hands respectively, and yielded a markedly improved diagnostic rate compared with mSDL or mMDL alone.

All NCS parameters were more often abnormal in obese patients. The mean BMI was greater in the cases with CTS than in the control. Slightly negative correlation was found between the BMI with mSCNV, mSNAPa and mMNCV. Also, there was a positive correlation between the BMI and mMDL. These data confirm the presence of a higher BMI in CTS and also show an increased risk of CTS with higher BMI. Obese populations are especially susceptible, given that CTS is connected with an increased BMI [12]. The relationship between BMI and CTS could be explained either due to increased fat deposition in the carpal canal or higher hydrostatic pressure in the carpal tunnel in obese subjects [13]. The sensitivity of the D4M-D4U, mSDL, mMDL and mTLI were 81.8%, 80%, 70.2% and 72.6% in obese patients, and 73%, 71%, 65.4% and 67.9% in non-obese patients, respectively. However, statistically significant differences were not found in sensitivities of NCSs parameters between two groups. These findings may be speculated that the obese patients had severe CTS and high sensitivity of parameter.

In conclusion, the newer nerve conduction techniques and combining different NCSs tests are more sensitive for the diagnosis of CTS. They may prove a useful addition in suspected cases and should increase diagnostic sensitivity when used in combination with conventional NCSs tests.

## Abbreviations

CTS: carpal tunnel syndrome; BMI: body mass index; NCSs: nerve conduction studies; mSNAPa: median sensory nerve action potential amplitude; mSDL: median sensory distal latency; mSNCV: sensory nerve conduction velocity; D4M-D4U: difference between the median and ulnar sensory distal latency to the fourth digit; D2M-D5U: difference between sensory median distal latency to second digit and ulnar distal latency to the fifth digit; D2M/U5 SNAPa ratio: median-to-ulnar SNAP amplitude ratio; mMDL: median motor distal latency; mMNCV: median motor nerve conduction velocity; mCMAPa: median compound muscle action potential amplitude; mTLI: median terminal latency index; M-U LD: the median-thenar to ulnar-hypothenar motor latency differences; M/U CMAPa ratio: the median-thenar to ulnar-hypothenar motor CMAPa ratio.

## Competing interests

The authors declare that they have no competing interests.

## Authors' contributions

RA, HU and DK played role in clinical evaluation and design of the study; RA and HU conducted electromyography; RD and MK performed the statistical analysis. All authors read and approved the final.
